# Efficacy of corticosteroid therapy for oxygen-free coronavirus disease 2019-derived pneumonia

**DOI:** 10.1097/MD.0000000000038932

**Published:** 2024-07-12

**Authors:** Zentaro Saito, Osamu Kanai, Natsumi Okamoto, Isao Watanabe, Mitsuhiro Tsukino

**Affiliations:** a Divison of Respiratory Medicine, Hikone Municipal Hospital, Hikone City, Japan; b Division of Respiratory Medicine, National Hospital Organization Kyoto Medical Center, Kyoto, Japan.

**Keywords:** corticosteroids, COVID-19, inverse probability of treatment weighting, pneumonia, SARS-CoV-2

## Abstract

Corticosteroid therapy for oxygen-free coronavirus disease 2019 (COVID-19) is not recommended due to its negative prognostic impact, but the efficacy of corticosteroids when limited to COVID-19 pneumonia is unclear. We aimed to evaluate the efficacy of corticosteroid monotherapy for patients with COVID-19 pneumonia without supplemental oxygen. We retrospectively reviewed patients with oxygen-free COVID-19 pneumonia at our institute between September 2020 and August 2021 and assessed the use of corticosteroids and the timing of initiation. We classified the patients into the following 2 groups: those who were initiated corticosteroids without developing respiratory failure (early steroid group) and those who were not (standard of care [SOC] group). We used inverse probability of treatment weighting (IPW) to balance between the groups. The primary outcome was the incidence of respiratory failure. A total of 144 patient records were reviewed; 63 patients were in the early steroid group and 81 patients were in the SOC group. Of all patients, 14 (22.2%) and 27 (33.3%) patients in the early steroid and SOC group, respectively, required supplemental oxygen (*P* = .192). After adjusted by the IPW method, 10 (16.0%) and 32 (40.1%) patients in the early steroid and SOC groups, respectively, required supplemental oxygen (*P* = .004). The logistic regression analysis indicated that early corticosteroid use was significantly associated with a decreased incidence of respiratory failure (odds ratio; 0.17, 95% confidence intervals; 0.06–0.46, *P* < .001). Corticosteroid monotherapy may suppress the development of exacerbation requiring oxygen supply in patients with oxygen-free COVID-19 pneumonia.

## 1. Introduction

Since severe acute respiratory syndrome coronavirus 2 (SARS-CoV-2) was first discovered in China in late 2019, coronavirus disease 2019 (COVID-19) has become a global pandemic.^[1]^ The COVID-19 pandemic has had serious medical, social, and economic impacts worldwide. As of May 2024, over 700 million people were confirmed to have a diagnosis of COVID-19 worldwide, and approximately 7 million patients with COVID-19 have died.^[2,3]^ Even after recovering from acute COVID-19, a variety of complications may remain for months or years.^[4]^ Some of the complications are severe enough to cause persistent disability.^[5]^

Currently, there are a variety of drugs for the treatment of COVID-19, including antiviral drugs, neutralizing antibody drugs, and immunosuppressive drugs.^[6–9]^ Corticosteroids exert anti-inflammatory effects by suppressing the production of many cytokines.^[10]^ Dexamethasone is a type of corticosteroid with anti-inflammatory properties that is less expensive than other COVID-19 therapeutics and is widely used around the world. It is approved by the U.S. Food and Drug Administration and the Ministry of Health, Labor and Welfare in Japan under COVID-19 as an effective drug for severe COVID-19.^[11]^ In the RECOVERY study, patients who received dexamethasone had a reduced 28-day mortality compared with patients who received standard therapy.^[12]^ In this trial, 21.5% of patients in the dexamethasone group who required oxygen died within 28 days of enrollment, compared with 25.0% of the control group. Several studies also provide support for the efficacy of systemic corticosteroid therapy in cases of moderate to severe COVID-19.^[13–17]^ Nevertheless, no prognostic benefit was observed in the population that did not require oxygen administration.^[12]^ Additionally, National Institutes of Health recommend against the use of dexamethasone in patients not requiring hospitalization or supplemental oxygen because patients with COVID-19 who did not require supplemental oxygen had an increased risk of 90-day mortality.^[18,19]^

Despite the recommendations set forth by the National Institutes of Health, we sometimes use corticosteroids for patients with COVID-19 pneumonia who do not require oxygen in the real world and experience clinical improvement. Therefore, we hypothesized that corticosteroid monotherapy would be beneficial in a particular group of COVID-19 patients without oxygenation. In the present study, we retrospectively evaluated the effect of corticosteroid monotherapy for patients with COVID-19 pneumonia who did not receive oxygen therapy.

## 2. Methods

### 2.1. Patients’ selection

This retrospective cohort study was conducted at Hikone Municipal Hospital, Shiga, Japan between September 2020 and August 2021. In Japan, the proportion of B.1.1.7 (alpha) variant was about 80% nationwide in May 2021, and B.1.617.2 (delta) variant were estimated to have accounted for about 85% of the total in August 2021.^[20,21]^ We identified patients with COVID-19 pneumonia who did not receive oxygen therapy (corresponding to a score of 3 on the WHO COVID ordinal outcome scale) on admission to our institute and retrospectively reviewed the clinical data of patients with COVID-19.^[22]^ Patients with SpO_2_ level below 95% in ambient air were excluded from the study as they require oxygenation. SARS-CoV-2 was detected using polymerase chain reaction or antigen tests to confirm the COVID-19 diagnosis. COVID-19 pneumonia was confirmed using chest radiography or computed tomography upon admission. As the included patients with COVID-19 pneumonia did not require oxygenation, most cases presented with mild pneumonia. Hence, we did not conduct radiographic scoring, and the diagnosis of COVID-19 pneumonia was solely based on the presence of any radiological findings. Due to the status of treatments applicable to COVID-19 in Japan, as described below, only antibody cocktail therapy could be used for mild COVID-19 during the study period. Firstly, remdesivir was only available to the COVID-19 patients with respiratory failure in Japan. Secondly, baricitinib was approved to the treatment for COVID-19 with respiratory failure in April 2021. Finally, the combination of casirivimab with imdevimab was approved for mild COVID-19 in July 2021. Thus, we excluded patients who received any neutralizing antibodies therapy.

### 2.2. Baseline characteristics

We obtained the following baseline clinical data of patients from the electrical medical record: age upon admission, sex, body mass index (BMI), smoking history, risk factors for the exacerbation of COVID-19, symptoms of COVID-19, including dyspnea and fever, defined as an axillary temperature of 37.5 degrees Celsius or over, the number of days from the onset of any COVID-19 symptoms to admission, transcutaneous oxygen saturation (SpO_2_) score, severe comorbidities of COVID-19, and blood and serum sample test results. The risk factors for the exacerbation of COVID-19 consisted of age 65 or over, obesity defined as a BMI score over 30 kg/m^2^, comorbidity with cardiovascular, metabolic, respiratory, kidney, or liver diseases, and immunosuppression.^[23,24]^ The blood and serum sample test results included white blood cell count, lymphocyte count, hemoglobin, platelet count, C-reactive protein (CRP), and lactate dehydrogenase (LDH). A history of vaccination against SARS-CoV-2 was not investigated because the vaccination coverage in Japan was as low as 15% in August 2021 due to the delay in the dissemination of vaccination.^[25,26]^

### 2.3. Grouping

We assessed the use of corticosteroids as a treatment for COVID-19 and the timing of its initiation. Corticosteroid therapy was initiated at the discretion of the attending physician. To evaluate the impact of early initiation of corticosteroid therapy for COVID-19, we classified the patients into the following 2 groups: those who were initiated corticosteroids without developing respiratory failure (early steroid group) and those who did not (standard of care [SOC] group). While those in the early steroid group were administered 6 mg of dexamethasone daily for 5 to 10 days from initiation, SOC group received corticosteroid therapy when the patient developed respiratory failure and started oxygenation.

### 2.4. Outcome

The primary outcome was the incidence of respiratory failure requiring oxygen supply within 14 days from symptom onset. The secondary outcome was the incidence of severe respiratory failure requiring invasive mechanical ventilation within 14 days from symptom onset. We also evaluated hyperglycemia and infections newly occurred during admission as potential adverse events associated with corticosteroid therapy. Hyperglycemia was defined as an increase in fasting blood glucose to 200 mg/dL or higher in patients without a history of diabetes. Infections were defined as newly occurring bacterial infections during admission.

### 2.5. Statistical analysis

The baseline characteristics of the patients were described in accordance with early steroid therapy. Continuous variables are presented as the mean and standard deviation (SD), and categorical variables are presented as counts and percentages. Similarity between the groups was examined by standardized mean differences (SMDs). A score of less than 0.1 in SMD was considered to be balanced. The incidence of oxygen supply and invasive mechanical ventilation were evaluated by Fisher’s exact test as a univariable analysis and by logistic regression analysis as a multivariable analysis. In the multivariable analysis, we included the number of risk factors for exacerbation of COVID-19, sex, fever, SpO_2_ upon admission, and the number of days from the onset of symptoms to admission, and then we optimized the logistic regression model by using the stepdown method based on Akaike’s information criterion.

We conducted propensity score (PS) analysis to evaluate the association between early steroid use and oxygen supply or invasive mechanical ventilation. We estimated the PS for each patient by using logistic regression with potential determinants as independent variables and early steroid use as the dependent variable. The regression model contained the following covariates: age, sex, obesity, current or former habit of smoking, having any high-risk factors for exacerbation of COVID-19, dyspnea, fever, days from symptom onset to admission, SpO_2_ score, and sample tests including lymphocyte fraction, platelet, CRP, and LDH. The validity of the logistic regression model was evaluated by a c-statistic score.

When evaluating the effect of early steroid therapy on outcomes, we estimated the average treatment effect stabilized by the prevalence of early steroid group (*P*_e_) using the following formula.


$$Stabilized\;ATE=\{\begin{array}{c} \;\frac{P_{e}}{PS}\;\text{(early sterod group)}\\ \frac{1-P_{e}}{1-PS}\;\text{(SOC group)} \end{array}$$

To address the potential selection bias, differences in baseline characteristics between the groups were controlled by using an IPW-adjusted analysis method. The covariate balance between the groups before and after IPW adjustment was assessed by SMD.

Sensitivity analysis to account for unmeasured confounders was performed by assessing an E-value, which was estimated by using the odds ratio (OR) obtained from the logistic regression as follows.^[27]^


$$E-Value=\{\frac{\sqrt{OR}+\sqrt{\sqrt{OR}\times \left(\sqrt{OR}-1\right)},OR\geq 1}{\sqrt{\frac{1}{OR}}+\sqrt{\sqrt{\frac{1}{OR}}\times \left(\sqrt{\frac{1}{OR}}-1\right)},OR<1}$$

Statistical tests with a two-sided *P* value of less than .05 as statistically significant, with 95% confidence intervals (CIs), were conducted. All statistical analyses were performed using R version 4.0.3 (R Foundation for Statistical Computing, Vienna, Austria).

### 2.6. Ethics

This study complied with the principles of the World Medical Association Declaration of Helsinki. The study protocol was approved by the Ethics Committee and Institutional Review Board of Hikone Municipal Hospital [approval number: 2021-06]. The results were reported in accordance with the Strengthening the Reporting of Observational Studies in Epidemiology statement. We have no conflicts of interest to report concerning this study.

## 3. Results

**Figure 1.**
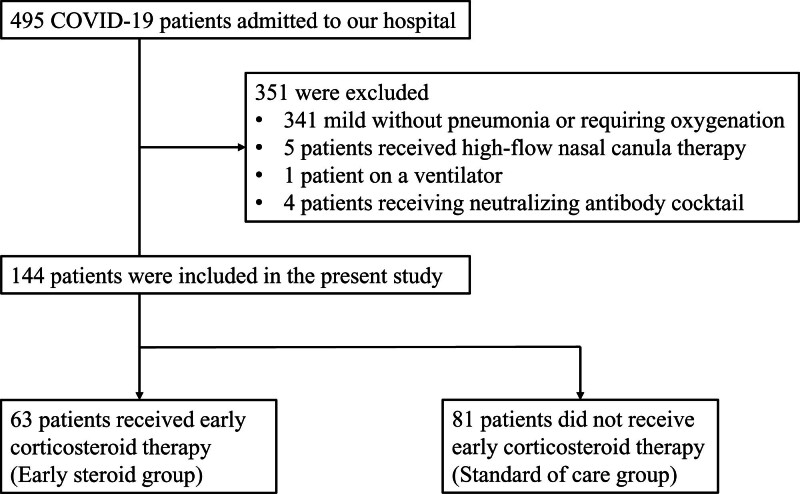
Study flowchart illustrating the process of screening, patient selection, exclusion, and classification. One patient who required ventilator care due to a preexisting neurological disorder was already dependent on a ventilator. The decision regarding the classification, that is, the initiation of corticosteroid therapy was made based on the judgement of the attending physician.

Between September 2020 and August 2021, 149 patients with COVID-19 pneumonia were admitted to Hikone Municipal Hospital. Of these 149 patients, one patient who required ventilator care due to a neurological disorder upon admission and 4 patients treated with the combination of casirivimab with imdevimab were excluded. Finally, a total of 144 patients were included in the study (Fig. 1). None of patients presented with complicating respiratory diseases such as asthma attack, acute exacerbation of chronic obstructive pulmonary disease, or bacterial pneumonia upon admission. Moreover, no patient had any severe complications, including Guillan-Barré syndrome. The unweighted baseline characteristics of the patients with COVID-19 are described in Table 1. Participants were classified into an early steroid group (63 patients) and a SOC group (81 patients). The mean ages were 51 and 55 years in the early steroid group and SOC group, respectively, with a SMD of 0.216. Additionally, fewer patients over 50 years of age were in the early steroid group than in the nonearly steroid group (29 [46.0%] vs 46 [56.8%], SMD = 0.217). There were 9 patients each in the early steroid and SOC groups (SMD = 0.095) with BMI values over 30 kg/m^2^. Of all the patients, 108 (75.0%) patients had at least one risk factor for the exacerbation of COVID-19. The prevalence of those with the risk factors (SMD = 0.049) and the number of risk factors (SMD = 0.026) were similar between the early steroid and SOC groups. Fifty-five (87.3%) and 43 (53.1%) patients in the early steroid and SOC groups, respectively, presented with fever, showing a large difference (SMD = 0.807). The mean SpO_2_ upon admission was lower in the early steroid group. (97.2% vs 97.6%, SMD = 0.482). The time from symptom onset to admission (SMD = 0.702) and time from symptom onset to the initiation of corticosteroid therapy (SMD = 1.114) in the early steroid group were 4.9 and 8.1 days, respectively, which were significantly longer than those for the SOC group. The sample tests conducted upon admission showed lower lymphocyte (SMD = 0.325) and platelet counts (SMD = 0.266) in the blood tests and higher CRP (SMD = 0.368) and LDH (SMD = 0.257) in the serum tests among the early steroid group.

**Table 1:** Unweighted baseline characteristics of the patients with COVID-19

Group	Early steroid		SOC		*P* value*	SMD
N	63		81			
Age (years)	51.4	(14.3)	55.0	(18.3)	.207	0.216
Generation						
Under 40	10	(15.9)	17	(21.0)	.096	0.488
40s	24	(38.1)	18	(22.2)		
50s	13	(20.6)	16	(19.8)		
60s	9	(14.3)	9	(11.1)		
70 or over	7	(11.1)	21	(25.9)		
Aged 65 or over	14	(22.2)	26	(32.1)	.260	0.223
Sex (male)	36	(57.1)	42	(51.9)	.614	0.106
Obesity†	9	(14.3)	9	(11.1)	.617	0.095
Current or former smoking	29	(46.0)	35	(43.2)	.739	0.057
Cardiovascular diseases	16	(25.4)	32	(39.5)	.079	0.305
Metabolic disorders	12	(19.0)	7	(8.6)	.084	0.305
Chronic respiratory diseases	7	(11.1)	7	(8.6)	.778	0.083
Kidney disfunction	4	(6.3)	2	(2.5)	.404	0.190
Liver dysfunction	4	(6.3)	4	(4.9)	.73	0.061
Under immunosuppression	5	(7.9)	6	(7.4)	>.99	0.020
Number of risk factor for exacerbation‡	1.4	(1.1)	1.4	(1.2)	.878	0.026
Having any risk factors‡	48	(76.2)	60	(74.1)	.847	0.049
Having any symptoms of COVID-19	62	(98.4)	68	(84.0)	.004	0.527
Dyspnea	6	(9.5)	8	(9.9)	>.99	0.012
Fever§	55	(87.3)	43	(53.1)	<.001	0.807
Symptom onset to admission (days)	4.9	(3.6)	2.8	(2.4)	<.001	0.702
SpO_2_ on admission (%)	97.2	(1.2)	97.6	(1.1)	.005	0.482
Disease onset to steroid (days)	8.1	(3.2)	5.2	(1.9)	<.001	1.114
Sample tests upon admission						
White blood cell (*10^3^/µL)	5.3	(1.8)	5.1	(1.6)	.461	0.123
Lymphocyte (*10^3^/µL)	1078.1	(529.1)	1252.7	(544.1)	.055	0.325
Lymphocyte fraction (%)	21.4	(8.9)	25.9	(10.7)	.009	0.447
Hemoglobin (g/dl)	14.5	(1.9)	13. 9	(2.2)	.089	0.291
Platelet (*10^4^/µL)	18.3	(6.3)	20.1	(7.3)	.119	0.266
C-reactive protein (mg/dL)	3.79	(4.2)	2.42	(3.2)	.027	0.368
Lactate dehydrogenase (IU/L)	241.1	(75.2)	221.4	(77.8)	.129	0.257

Data are shown with mean and standard deviation in continuous variables or number and percentage in categorical variables.

SMD = standardized mean difference, SOC = standard of care.

* *P* values are estimated by *t* tests for continuous variables or Fisher’s exact tests for categorical variables.

† Obesity is defined as a body mass index over 30 kg/m^2^.

‡ Risk factors for exacerbation of COVID-19 are defined as the follows: over 50 years of age, obesity, comorbidity with cardiovascular disease, metabolic disorders, chronic respiratory diseases, kidney dysfunction, liver dysfunction, or under immunosuppression.

§ Fever is defined as an axillary temperature of 37.5 degrees Celsius or higher.

To improve the balance of baseline characteristics between the groups, we performed an IPW-adjusted analysis. Table 2 shows the results of the logistic regression analysis performed in the process of estimating PS. The logistic model employed for PS estimation yielded a c-statistic of 0.812, affirming the validity of the model. According to this analysis, patients presenting with fever, a longer time from symptom onset to admission, and lower SpO_2_ level upon admission were more prone to be classified into the early steroid group. Table 3 shows weighted baseline characteristics of the patients with COVID-19. The number of factors above 0.1 in the SMD decreased from 19 to 7 items out of 25 after IPW adjustment, improving the balance between the groups.

**Table 2:** Logistic regression analysis for early steroid use.

	OR	95% CI	*P* value
Age (years)	0.99	(0.95–1.02)	.375
Sex (Male)	0.89	(0.34–2.33)	.807
Obesity	0.87	(0.21–3.62)	.853
Current or former smoking	1.22	(0.50–2.95)	.661
Having any high-risk factors	1.35	(0.41–4.50)	.625
Dyspnea	0.73	(0.17–3.20)	.679
Fever	4.80	(1.70–13.60)	.003
Symptom onset to admission (days)	1.27	(1.10–1.48)	.001
SpO_2_ on admission (%)	0.60	(0.41–0.90)	.013
White blood cell (*10^3^/µL)	0.99	(0.74–1.33)	.965
Lymphocyte fraction (%)	0.97	(0.92–1.01)	.154
Hemoglobin (g/dL)	1.00	(0.77–1.29)	.987
Platelet (*10^4^/µL)	0.94	(0.87–1.02)	.121
C-reactive protein (mg/dL)	0.99	(0.86–1.13)	.847
Lactate dehydrogenase (IU/L)	1.00	(0.99–1.00)	.291

Odds ratios and 95% CIs for early steroid use are estimated by the logistic regression analysis. We estimated the propensity score for grouping based on the analysis.

CI = confidence interval, OR = odds ratio.

**Table 3:** Weighted baseline characteristics of the patients with COVID-19.

Group	Early steroid		SOC		*P* value*	SMD
N (Adjusted)	64		79			
Age (years)	54.8	(15.6)	53.0	(17.5)	.615	0.110
Generation						
Under 40	9	(13.4)	19	(24.5)	.570	0.367
40s	22	(34.0)	20	(25.6)		
50s	12	(18.9)	15	(19.6)		
60s	10	(15.6)	7	(8.9)		
70 or over	12	(18.1)	17	(21.4)		
Aged 65 or over	21	(32.8)	21	(26.6)	.606	0.117
Sex (Male)	35	(55.2)	43	(54.6)	.957	0.012
Obesity†	8	(12.2)	12	(15.5)	.626	0.095
Current or former smoking habit	27	(41.8)	30	(38.2)	.729	0.072
Cardiovascular diseases	16	(25.6)	28	(35.1)	.355	0.207
Metabolic disorders	12	(18.0)	7	(9.2)	.179	0.26
Chronic respiratory diseases	5	(7.8)	10	(12.8)	.401	0.167
Kidney disfunction	3	(4.7)	2	(2.6)	.483	0.114
Liver dysfunction	4	(5.5)	3	(3.9)	.648	0.072
Under immunosuppression	4	(6.6)	5	(6.8)	.956	0.010
Number of risk factor for exacerbation	1.3	(1.1)	1.4	(1.1)	.890	0.026
Having any risk factors‡	47	(74.4)	61	(77.5)	.728	0.071
Having any symptoms of COVID-19	58	(91.8)	69	(88.0)	.695	0.125
Dyspnea	4	(5.8)	6	(7.6)	.626	0.072
Fever§	45	(70.2)	53	(67.6)	.816	0.057
Symptom onset to admission (days)	3.5	(3.3)	3.3	(2.6)	.728	0.067
SpO_2_ on admission (%)	97.4	(1.1)	97.4	(1.1)	.894	0.025
Onset to steroid (days)	8.1	(3.0)	5.8	(2.1)	NA	0.910
Sample tests on admission						
White blood cell (*10^3^/µL)	5.2	(1.7)	5.2	(1.6)	.824	0.045
Lymphocyte (*10^3^/µL)	1155.6	(465.9)	1188.5	(529.3)	.713	0.066
Lymphocyte fraction (%)	23.6	(8.7)	23.8	(10.7)	.883	0.028
Hemoglobin (g/dL)	14.2	(2.3)	14.2	(2.2)	.990	0.003
Platelet (*10^4^/µL)	18.6	(7.3)	19.0	(6.5)	.806	0.054
C-reactive protein (mg/dL)	3.2	(3.5)	2.9	(3.2)	.642	0.083
Lactate dehydrogenase (IU/L)	227.2	(73.5)	230.3	(81.1)	.836	0.040

Data are shown with mean and standard deviation in continuous variables or number and percentage in categorical variables. The inverse probability weighting (IPW) method is used to adjust the differences between the groups.

NA = not applicable, SMD = standardized mean difference, SOC = standard of care.

* *P* values are estimated by t tests for continuous variables or Fisher’s exact tests for categorical variables.

† Obesity is defined as a body mass index over 30 kg/m^2^.

‡ Risk factors for exacerbation of COVID-19 are defined as the follows: over 50 years of age, obesity, comorbidity with cardiovascular disease, metabolic disorders, chronic respiratory diseases, kidney dysfunction, liver dysfunction, or under immunosuppression.

§ Fever is defined as an axillary temperature of 37.5 degrees Celsius or higher.

Table 4 shows unweighted univariable analysis for outcomes. Fourteen (22.2%) and 27 (33.3%) patients required oxygen supply in the early steroid and SOC groups, respectively, but the difference was not significant (*P* = .192). Additionally, 1 (1.6%) and 4 (4.9%) patients required tracheal intubation in the early steroid and SOC groups, respectively (*P* = .386). The clinical features of patients who experienced respiratory failure are shown in Table S1 (Supplemental Digital Content, http://links.lww.com/MD/N199). Noteworthy risk factors associated with the development of respiratory failure included higher age, the presence of any exacerbation-related risk factors (particularly cardiovascular and liver diseases), fever, and lower SpO_2_ upon admission, In the weighted univariable analysis, the number of patients requiring oxygen was 10 (16.0%) in the early steroid group and 32 (40.1%) in the SOC group, which shows a significant difference (*P* = .004). Additionally, 1 (1.4%) and 5 (5.9%) patients in the early steroid and SOC groups, respectively, required tracheal intubation (*P* = .171).

**Table 4:** Unweighted and weighted univariable analysis for outcomes.

Group	Unweighted						Weighted					
	Early steroid		SOC		*P* value	SMD	Early steroid		SOC		*P* value	SMD
N	63						64		79			
Oxygen supply	14	(22.2)	27	(33.3)	.192	0.250	10	(16.0)	32	(40.1)	.004	0.556
Mechanical ventilation	1	(1.6)	4	(4.9)	.386	0.189	1	(1.4)	5	(5.9)	.171	0.240
Corticosteroid use	63	(100.0)	27	(33.3)	<.001	2.000	16	(25.2)	22	(27.4)	.812	0.050
Corticosteroids-associated adverse events												
Hyperglycemia	20	(31.7)	15	(18.5)	.079	0.309	16	(25.2)	21	(26.4)	.902	0.026
Infections	2	(3.2)	4	(4.9)	.696	0.089	2	(2.6)	5	(5.9)	.332	0.166

*P* values are estimated by Fisher’s exact tests. In the weighted analysis, differences between the groups are adjusted by the IPW method.

IPW = inverse probability treatment weighting, SMD = standardized mean difference.

Table 5 shows the results of multivariable analyses for respiratory failure due to COVID-19 pneumonia. The unweighted analysis performed during the final step shows that early corticosteroid use was significantly associated with a decreased incidence of respiratory failure (OR: 0.23; 95% CI: 0.09–0.62, *P* value = .003) (Table 5). The weighted analysis performed during the final step indicated the early corticosteroid use was also significantly associated with a decreased incidence of respiratory failure (OR: 0.17; 95% CI: 0.06–0.46, *P* value < .001) (Table 6).

**Table 5:** Unweighted multivariable analysis for requiring oxygen supply.

Factor	First step			Final step		
	OR	95% CI	*P* value	OR	95% CI	*P* value
Early corticosteroid use	0.27	(0.10–0.75)	.012	0.23	(0.09–0.62)	.003
Sex (male)	1.51	(0.62–3.70)	.370			
Number of risk factors	1.96	(1.32–2.93)	<.001	2.05	(1.39–3.02)	<.001
Fever	4.55	(1.57–13.20)	.005	4.36	(1.53–12.40)	.006
Symptom onset to admission	0.93	(0.79–1.08)	.330			
SpO_2_ on admission	0.59	(0.39–0.89)	.012	0.56	(0.37–0.83)	.004

Odds ratios and 95% CIs for requiring oxygen supply are estimated by the logistic regression analysis. The first step column shows the logistic regression analysis results with all six factors as independent variables. The final step column shows the logistic regression analysis results optimized by the stepdown method.

CI = confidence interval, OR = odds ratio.

**Table 6:** Weighted multivariable analysis for requiring oxygen supply.

Factor	First step			Final step		
	OR	95% CI	*P* value	OR	95% CI	*P* value
Early corticosteroid use	0.18	(0.07–0.48)	<.001	0.17	(0.06–0.46)	<.001
Sex (male)	1.26	(0.50–3.17)	.630			
Number of risk factors	1.79	(1.17–2.75)	.008	1.86	(1.23–2.83)	.003
Fever	6.23	(1.91–20.30)	.003	5.98	(1.85–19.30)	.003
Symptom onset to admission	0.94	(0.79–1.11)	.440			
SpO_2_ on admission	0.50	(0.32–0.76)	.001	0.48	(0.32–0.73)	<.001

Odds ratios and 95% CIs for requiring oxygen supply are estimated by logistic regression analysis. The first step column shows the logistic regression analysis results with all 6 factors as independent variables. The final step column shows the logistic regression analysis results optimized by the stepdown method.

CI = confidence interval, OR = odds ratio.

We obtained an E-value of 4.28 for early corticosteroid therapy based on the results of optimized logistic regression analysis weighted by the IPW method. The association between early corticosteroid therapy and the development of respiratory failure could be canceled only by an unmeasured confounder that is associated with both early steroid therapy and the development of respiratory failure by a risk ratio of 4.28-fold above the measured confounders.

## 4. Discussion

In this retrospective study, we observed a lower incidence of oxygen therapy in the early steroid group compared to the SOC group. To date, there exists solitary study that assess the efficacy of corticosteroid monotherapy limited to mild patients with COVID-19 including pneumonia without oxygenation.^[28]^ This randomized controlled study demonstrated a 7.53% reduction in the risk of oxygenation requirement with the use of corticosteroids. In the study, approximately two-thirds of the participants presented with COVID-19 pneumonia but did not require oxygenation. However, these results were the opposite of those reported in the past, suggesting that corticosteroid monotherapy rather prevented exacerbations. The Veterans Aging Cohort Study Clinical COVID-19 Working Group showed that corticosteroid was associated with increased mortality in patients with no oxygen administration.^[19]^ The RECOVERY study showed a poor benefit of dexamethasone for patients not on oxygen.^[12]^ However, in the former study, most of the participants were male and hospitalized within a day of being diagnosed with COVID-19, and the time from onset was unknown.^[19]^ Although the presence of COVID-19 pneumonia was not investigated in those studies, it is suspicious that the time from symptom onset to administration of corticosteroids in mild COVID-19 patients may be shorter than that in moderate to severe patients. In a study of 44,672 patients with COVID-19 in China, 81% of patients with mild disease are cured within a week, and 14% of patients become severe within 1 week to 10 days, and 5% develop critical manifestations thereafter.^[29]^ COVID-19 pneumonia is considered a sign before the disease begins to become severe, and corticosteroid administration at certain times of pneumonia may be beneficial.^[28]^

In general, COVID-19 has a viral response phase and a host inflammatory response phase.^[30]^ Viral shedding peaks earlier in COVID-19 compared to past coronavirus infections such as middle east respiratory syndrome and severe acute respiratory syndrome (SARS).^[31]^ According to this previous report, peak viral shedding is 2.0 days for COVID-19, 7.2 days for SARS, and 12.2 days for middle east respiratory syndrome. Also, a previous study of SARS found that corticosteroid therapy may delay virus clearance and be associated higher viral concentrations.^[32]^ On the other hand, some studies reported that the administration of corticosteroid to patients with COVID-19 may not affect clearance of SARS-CoV-2.^[33,34]^ In COVID-19 REGISTRY JAPAN study, the median time from COVID-19 onset to hospitalization is 7 days, by which time the peak of viral shedding is expected to have already passed, suggesting that this may be the optimal period for the effectiveness of corticosteroids.^[35]^ In the present study, the duration from onset to steroid administration was 8.1 days in the early steroid group, indicating that patients in this group received corticosteroid treatment during the appropriate inflammatory phase to prevent the exacerbation of COVID-19 pneumonia.

Hyperbaric oxygen therapy (HBOT) may help manage the inflammatory phase of COVID-19 while avoiding the delayed viral shedding caused by corticosteroids.^[36,37]^ Recently, HBOT has been reported to decrease inflammatory responses to SARS-CoV-2, including serum levels of CRP, ferritin, and LDH in a small randomized controlled study.^[38]^ In contrast, HBOT has 2 limitations; the one is that the efficacy of HBOT on the severity of respiratory failure in COVID-19 has not been confirmed prospectively.^[38,39]^ The other is that most institutes have limited access to HBOT, including our hospital. Thus, there was no patient who underwent HBOT in the present study.

In this study, approximately 25% of patients had no risk factor for the exacerbation of COVID-19. While neutralizing antibodies or antiviral drugs may be used for at-risk patients, the reality is that there are few treatment options for patients who are not at risk, even if they have a COVID-19 pneumonia.^[6–9]^ Corticosteroids are used for decades to treat a variety of diseases, including interstitial pneumonia, rheumatic diseases, and nephrotic syndrome. In the face of a pandemic, corticosteroids may be a safe drug to use around the world, including in developing countries.

This study has some limitations. First, due to the retrospective nature of the study, this study cannot explain the causality of the beneficial effect of early corticosteroid use on the treatment of COVID-19 pneumonia. Although the causality would be proven by a prospective randomized-controlled study, the study comparing the effect of corticosteroid monotherapy to placebo is ethically unacceptable. This is because now several antiviral agents and neutralizing antibodies are available in the treatment of mild COVID-19 with the risk factors of exacerbation.^[6–9]^ Second, this study included an insufficient number of patients to perform a multivariate analysis. Thus, we could not add a variety of factors associated with the prognosis of COVID-19, including lymphocyte count, CRP, and LDH.^[23,40,41]^ Third, the baseline characteristics of each group were not well balanced because the patients were not randomly allocated to each group. For solving these biases, we conducted IPW adjusted analysis. The balance of the factors, which was not included in the multivariate analysis including the sample test results, was improved by adjusting with the IPW method. Fourth, generalizability of the results was not fully warranted because this study was a single-center study and consisted of Asian ethnic patients only. Furthermore, not all COVID-19 pneumonia patients in the lesion admitted to our institute. The patients with risk factors for exacerbation and with symptoms such as fever and dyspnea were admitted preferentially; however, these factors were adjusted on the multivariable analysis. Even if there was an unmeasured bias between the patients admitted to our hospital and outpatients or between early steroid group and non-early steroid group, the sensitivity analysis indicated that the unmeasured bias which diminish the association of early corticosteroid use and fewer incidence of respiratory failure should have a risk ratio of 4.28 or above. We could not envision any unmeasured bias with such a high-risk ratio other than antiviral drugs.^[6–9]^ Finally, the results of this study may not be directly applicable to current COVID-19 practice; to date, antiviral drugs for mild COVID-19 and vaccines for SARS-CoV-2 are common in clinical practice.^[8,9,42]^ Conversely, the results of this study show the effect of corticosteroids alone, with the effects of antivirals and vaccines excluded. In contrast, corticosteroids and immunomodulators are still reported to be effective to COVID-19 pneumonia even in antiviral drugs and vaccination era. A randomized controlled study showed that abatacept and infliximab showed significantly low mortality within 28 days.^[43]^ Moreover, high dose corticosteroids show comparable efficacy to the standard dose in COVID-19 pneumonia with earlier improvement of symptoms.^[44]^ From these recent studies, immunosuppressive agents may have preferable effects on COVID-19 pneumonia. Although there are several limitations, it was a remarkable result corticosteroid monotherapy could have a preventive effect on severe progression.

## 5. Conclusions

Corticosteroid monotherapy for patients with COVID-19 pneumonia without need for oxygen supply can be a beneficial treatment option. The presence of radiographic evidence of pneumonia could potentially serve as a pivotal factor in distinguishing whether corticosteroid therapy offer a beneficial or harmful effect on oxygen-free COVID-19 patients. Further study investigating the efficacy of corticosteroids for COVID-19 pneumonia is warranted, including the efficacy of a combination of corticosteroid and anti-viral therapy for oxygen-free COVID-19 pneumonia.

## Acknowledgments

We would like to thank American Journal Experts (https://www.aje.com) for their help with English language editing.

## Author contributions

**Conceptualization:** Zentaro Saito, Osamu Kanai, Isao Watanabe, Mitsuhiro Tsukino.

**Data curation:** Zentaro Saito, Natsumi Okamoto, Isao Watanabe, Mitsuhiro Tsukino.

**Formal analysis:** Osamu Kanai.

**Investigation:** Zentaro Saito, Natsumi Okamoto.

**Methodology:** Osamu Kanai, Mitsuhiro Tsukino.

**Project administration:** Zentaro Saito.

**Resources:** Zentaro Saito, Natsumi Okamoto, Isao Watanabe, Mitsuhiro Tsukino.

**Software:** Osamu Kanai.

**Supervision:** Osamu Kanai, Isao Watanabe, Mitsuhiro Tsukino.

**Validation:** Osamu Kanai.

**Writing – original draft:** Zentaro Saito.

**Writing – review & editing:** Osamu Kanai.

## Supplementary Material


