# Supplementary material for: Efficacy of corticosteroid therapy for oxygen-free coronavirus disease 2019-derived pneumonia
Source: Medicine (Baltimore). 2024 Jul 12;103(28):e38932. doi: 10.1097/MD.0000000000038932 (PMC11245202; doi:10.1097/MD.0000000000038932)
Supplement: Supplementary file 1 [file medi-103-e38932-s001.docx]

| Table S1: Baseline characteristics classified by the development of respiratory failure. | | | | | | |  |
| --- | --- | --- | --- | --- | --- | --- | --- |
| Development of respiratory failure |  | Yes | | No | | P value^1^ | SMD^*^ |
| N |  | 41 |  | 103 |  |  |  |
| Age | (Years) | 62.9 | (16.2) | 49.7 | (15.5) | <0.001 | 0.831 |
| Generation | Under 40 | 2 | (4.9) | 25 | (24.3) | 0.004 | 0.765 |
|  | 40s | 10 | (24.4) | 32 | (31.1) |  |  |
|  | 50s | 9 | (22.0) | 20 | (19.4) |  |  |
|  | 60s | 5 | (12.2) | 13 | (12.6) |  |  |
|  | 70 or over | 15 | (36.6) | 13 | (12.6) |  |  |
| Aged 65 or over |  | 20 | (48.8) | 20 | (19.4) | 0.001 | 0.651 |
| Sex | (Male) | 27 | (65.9) | 51 | (49.5) | 0.096 | 0.335 |
| Obesity^2^ |  | 7 | (17.1) | 11 | (10.7) | 0.402 | 0.186 |
| Current or former smoking |  | 21 | (51.2) | 43 | (41.7) | 0.354 | 0.191 |
| Cardiovascular diseases |  | 22 | (53.7) | 26 | (25.2) | 0.002 | 0.608 |
| Metabolic disorders |  | 6 | (14.6) | 13 | (12.6) | 0.787 | 0.059 |
| Chronic respiratory diseases |  | 3 | (7.3) | 11 | (10.7) | 0.757 | 0.118 |
| Kidney disfunction |  | 4 | (9.8) | 2 | (1.9) | 0.055 | 0.338 |
| Liver dysfunction |  | 6 | (14.6) | 2 | (1.9) | 0.007 | 0.473 |
| Under immunosuppression |  | 7 | (17.1) | 4 | (3.9) | 0.013 | 0.441 |
| Number of risk factor for exacerbation^3^ |  | 2.1 | (1.3) | 1.1 | (10) | <0.001 | 0.803 |
| Having any risk factors^3^ |  | 37 | (90.2) | 71 | (68.9) | 0.014 | 0.548 |
| Having any symptoms of COVID-19 |  | 38 | (92.7) | 92 | (89.3) | 0.757 | 0.118 |
| Dyspnea |  | 4 | (9.8) | 10 | (9.7) | >0.99 | 0.002 |
| Fever^4^ |  | 33 | (80.5) | 65 | (63.1) | 0.049 | 0.394 |
| Symptom onset to admission | (Days) | 3.0 | (2.5) | 4.0 | (3.3) | 0.071 | 0.355 |
| SpO_2_ on admission | (%) | 96.9 | (1.1) | 97.6 | (1.1) | 0.001 | 0.622 |
| Onset to steroid | (Days) | 5.8 | (2.7) | 8.4 | (3.1) | <0.001 | 0.910 |
| White blood cell | (*10^3^/µl) | 5.3 | (1.8) | 5.1 | (1.6) | 0.358 | 0.165 |
| Lymphocyte | (*10^3^/µl) | 1052.3 | (511.6) | 1225.7 | (549.3) | 0.084 | 0.327 |
| Lymphocyte fraction | (%) | 20.6 | (8.8) | 25.2 | (10.4) | 0.013 | 0.481 |
| Hemoglobin | (g/dl) | 14.4 | (2.3) | 14.1 | (2.0) | 0.375 | 0.160 |
| Platelet | (*10^4^/µl) | 17.0 | (4.7) | 20.2 | (7.4) | 0.013 | 0.505 |
| C-reactive protein | (mg/dl) | 4.4 | (4.4) | 2.5 | (3.3) | 0.005 | 0.495 |
| Lactate dehydrogenase | (IU/l) | 265.9 | (91.0) | 215.8 | (66.0) | <0.001 | 0.630 |
| Early corticosteroids use |  | 14 | (34.1) | 49 | (47.6) | 0.192 | 0.276 |
| Data are shown with mean and standard deviation in continuous variables or number and percentage in categorical variables. | | | | | | | |
| 1. P values are estimated by t tests for continuous variables or Fisher’s exact tests for categorical variables. 2. Obesity is defined as a body mass index over 30 kg/m^2^. 3. Risk factors for exacerbation of COVID-19 are defined as the follows: over 50 years of age, obesity, comorbidity with cardiovascular disease, metabolic disorders, chronic respiratory diseases, kidney dysfunction, liver dysfunction, or under immunosuppression. 4. Fever is defined as an axillary temperature of 37.5 degrees Celsius or higher. | | | | | | | |
| * SMD; standardized mean difference. | | | | | | | |
